# SNX17 mediates STAT3 activation to promote hepatocellular carcinoma progression via a retromer dependent mechanism

**DOI:** 10.7150/ijbs.110506

**Published:** 2025-03-31

**Authors:** Yuqi Liu, Wei Tian, Chao Ge, Canxue Zhang, Zhihong Huang, Chi Zhang, Yue Yang, Hua Tian

**Affiliations:** 1 State Key Laboratory of Systems Medicine for Cancer, Shanghai Cancer Institute, Renji Hospital, Shanghai Jiao Tong University School of Medicine, Shanghai, China.; 2 Department of Pathology, The Affiliated Hospital of Youjiang Medical University for Nationalities, Baise 533000, China.; 3 The Key Laboratory of Molecular Pathology (Hepatobiliary Diseases) of Guangxi, Baise 533000, China.

**Keywords:** SNX17, STAT3, Oxidative phosphorylation, retromer, hepatocellular carcinoma

## Abstract

Endocytosis has emerged as a key regulator of malignant behavior in cancer. Members of the sorting nexin (SNX) family have been found to be dysregulated in various cancers and play significant roles in regulating tumor metastasis. However, the role and mechanism of SNX17 in hepatocellular carcinoma (HCC) progression remain largely unknown. Here, we found that upregulation of SNX17 in HCC was associated with poor prognosis. Overexpression of SNX17 promoted HCC cell proliferation, migration, invasion, and metastasis, whereas silencing SNX17 expression resulted in opposite effects. Knockdown of SNX17 induced G1/S phase arrest and apoptosis. We discovered that SNX17 directly interacted with STAT3 and increased its phosphorylation in a retromer-dependent manner. SNX17-retromer complex acted as a platform for IL-6-induced STAT3 activation. Activated STAT3 then increased c-Myc expression and promoted mitochondrial oxidative phosphorylation (OXPHOS) and mitochondrial biogenesis. SNX17 overexpression-induced OXPHOS was reversed by c-Myc inhibitor. Knockdown of STAT3 expression or treatment with a STAT3 inhibitor significantly attenuated SNX17-enhanced proliferation and invasion. Taken together, our results indicate that SNX17 promotes HCC cell proliferation and metastasis through direct interaction with STAT3 in a retromer-dependent manner, thereby activating the STAT3/c-Myc signaling pathway and enhancing OXPHOS. These findings suggest that SNX17 is a potential therapeutic target for HCC.

## Introduction

Hepatocellular carcinoma (HCC) is the fourth most common cause of cancer-related death and ranks sixth in incidence among cancers worldwide 1,2. Surgical resection is recognized as an effective treatment for early-stage HCC 3. Despite advances in molecular targeted therapies and immunotherapy over the past decade, the treatment outcomes for HCC are still not satisfactory 4-6. Therefore, a better characterization of oncogenic signaling pathways and tumor immunological regulation is essential to improve the prognosis of HCC patients.

Endocytosis is a fundamental process that regulates cellular metabolism, receptor internalization, signaling, and nutrient uptake 7. Therefore, endocytosis plays a crucial role in maintaining cell function and survival 8,9. The sorting nexins (SNXs) are a family of proteins that regulate cellular cargo sorting, endocytosis and trafficking. Dysfunctions in SNX proteins have been increasingly associated with tumor progression, highlighting their critical roles in cancer biology. For example, SNX10 has been shown to inhibit colorectal cancer initiation and progression by facilitating the autophagic degradation of SRC 10. Conversely, SNX10 sustains PDGF receptor signaling in glioblastoma stem cells 11. SNX27 promotes breast cancer metastasis 12. Our previous studies have shown that SNX5 promotes HCC progression through modulating the EGFR-ERK1/2 signaling pathway 13. However, SNX5 suppresses clear cell renal cell carcinoma progression by inducing CD44 internalization and epithelial-to-mesenchymal transition 14. As a member of the SNX family, SNX17 has been implicated in various physiological and pathological processes. SNX17 mediates atrial fibrillation and protects the heart from doxorubicin-induced cardiotoxicity 15,16. Additionally, SNX17 regulates synaptic function and plasticity 17. Despite these findings, the role of SNX17 in HCC remains poorly understood. Therefore, we aimed to explore the function of SNX17 in HCC and the molecular mechanisms underlying its role.

The signal transducer and activator of transcription (STAT) proteins family, comprising STAT1, STAT2, STAT3, STAT4, STAT5a, STAT5b and STAT6, mediates multiple intracellular signaling pathways and influences the initiation of malignant transformation and cancer progression 18. Among these, STAT3 is a key regulator of cell proliferation, survival differentiation, and angiogenesis. STAT3 is frequently hyperactivated and associated with poor clinical prognosis in the majority of human cancers, making it an important potential therapeutic target for cancer treatment 19,20. The phosphorylated STAT3 (Y705) forms a dimer that translocates to the nucleus and functions as a transcription factor 19. Previous study has shown that STAT3 is actively transported via receptor-mediated endocytosis 21. To date, it remains largely unknown whether the sorting nexins are involved in the phosphorylation and translocation of STAT3 in HCC.

## Materials and Methods

### Cell lines and cell culture

Huh7 and Hepa1-6 cells were obtained from Riken Cell Bank (Tsukuba, Japan). HEK-293T cell lines were purchased from the American Type Culture Collection (Manassas, VA, USA). The MHCC-LM3 and MHCC-97H cell lines were obtained from the Liver Cancer Institute, Zhongshan Hospital of Fudan University (Shanghai, China). The HCC-LY10 cell line was established in our laboratory. The HCC cell lines used in this study were cultured in Dulbecco's modified Eagle's medium (DMEM) (Gibco) containing 10% heat-inactivated fetal bovine serum (Gibco) and incubated at C in a humidified atmosphere with 5% CO_2_. All cell lines were authenticated and characterized by the suppliers, used within 6 months of resuscitation, and confirmed to be mycoplasma-free. Routine quality checks included verifying morphology and growth profiles to ensure the integrity of the cell lines.

### Lentivirus production and cell transduction

The SNX17 lentiviral overexpression plasmid and shRNA plasmid were supplied by the CCSB-Broad Lentiviral Expression Library and Human TRC shRNA Library. The plasmid was sequenced from the 5′ and 3′ ends to confirm its sequence. The STAT3 and c-Myc overexpression plasmids, along with the VPS35 knockdown plasmid, are maintained in our laboratory. The target sequences are listed in Supplementary Table S1. The mouse SNX17 lentiviral overexpression plasmid was constructed in our laboratory. Sequences of primers are listed in Supplementary Table S2.

Viral packaging was performed in HEK-293T cells by co-transfecting the SNX17 overexpression or shRNA plasmid along with the packaging plasmid psPAX2 and the envelope plasmid pMD2.G (Addgene) using Lipofectamine 2000 (Invitrogen). Viruses were harvested at 48 hours after transfection, and viral titers were determined. HCC cells were transduced with 1×10^6^ recombinant lentivirus-transducing units in the presence of 6 μg/ml polybrene (Sigma, USA).

### Quantitative real-time RT‒PCR (qRT‒PCR)

Total RNA extraction and reverse transcription were performed as previously described 20. qRT‒PCR analyses were conducted using an ABI Prism 7500 System (Applied Biosystems, Carlsbad, CA, USA) with SYBR® Premix Ex Taq (Takara, Dalian, China).

The mRNA levels were normalized to those of the housekeeping gene GAPDH. The sequences of the primers used in these experiments are listed in Supplementary Table S2.

### Western blotting

Proteins in whole cell lysates were separated by SDS‒polyacrylamide gel electrophoresis and transferred onto PVDF membranes (Millipore, USA). The membranes were incubated overnight with primary antibodies at 4 °C and then with secondary antibodies conjugated to horseradish peroxidase (HRP) for 1 hour at room temperature. The immunoreactive blots were visualized using an enhanced chemiluminescence reagent (Pierce, Rockford, IL, USA). β-Actin was used as a loading control. Detailed information on the antibodies used is provided in Supplementary Table S3.

### Cell proliferation and colony formation assays

Cell proliferation was measured by the Cell Counting Kit-8 (CCK-8) (Bimake, USA) according to the manufacturer's instructions. Briefly, cells were treated with CCK-8 solution and the absorbance was measured at 450 nm using a microplate reader.

The proliferation of cells was analyzed using an EdU Incorporation Assay Kit (Abbkine, Wuhan, China) according to the manufacturer's protocols. After EdU staining, the cells were observed and photographed using a fluorescence microscope.

For colony formation assays, HCC cells were seeded into 6-well plates at a density of 10^3^ cells/well and incubated at 37 °C for 2 weeks. Colonies were fixed with 4% phosphate-buffered formalin (pH 7.4) and subjected to Giemsa staining for 15 minutes. Each assay was performed in three separate experiments.

### Migration and invasion assays

Cells were seeded in the upper chamber of a transwell (8-μm pore size) or in a Matrigel-coated transwell insert (BD Biosciences, NJ) in serum-free media. The lower chamber contained DMEM supplemented with 10% fetal bovine serum as a chemoattractant. After incubating for 24 or 48 h, the non-migrated or non-invaded cells were gently removed from the upper chamber using a cotton swab. The remaining cells were fixed with formalin and stained with Giemsa solution. The number of cells in five randomly chosen fields of view was counted under a microscope.

### Flow cytometry analysis

For cell cycle analysis, cells were washed twice with cold PBS, fixed in 70% cold ethanol and incubated overnight. Before analysis, cells were stained with a solution containing 10 mg/ml RNAse and 400 mg/ml propidium iodide (PI) and incubated for 30 min at 37 °C. The stained cells were analyzed by flow cytometry and the data were analyzed using FlowJo software.

For the apoptosis assay, cells were harvested and washed with cold PBS. Cells were incubated with PE-conjugated Annexin V and 7-AAD for 15 minutes at room temperature. The cells were analyzed by flow cytometry, and the data were analyzed using FlowJo software. The results are the representative of 3 independent experiments with triplicate samples for each assay.

### In vivo growth and metastasis assays

To assess the in vivo growth and metastasis of HCC cells, four- to six- week-old male BALB/C nude mice used. HCC cells were orthotopically inoculated into the left hepatic lobes of the mice using a microsyringe through an 8-mm transverse incision in the upper abdomen under anesthesia. For the in vivo isograft experiment, male C57BL/6 mice were subcutaneously injected with Hepa1-6 derived cells. A total of 2 × 10^6^ cells suspended in 40 μl of a mixture of serum-free DMEM/Matrigel (1:1 volume) (BD Biosciences, MA) were injected into each nude mouse. The mice were sacrificed at six weeks post-inoculation, and the tissues were harvested and fixed with phosphate-buffered neutral formalin for at least 72 hours. Metastases were identified by analyzing lung tissue sections followed by H&E staining. All of the experiments were approved by the Renji Hospital Institutional Animal Care (RT2022-122u) and Use Committee and performed in accordance with the Institutional Guide for the Care and Use of Laboratory Animals.

### Immunohistochemistry (IHC)

IHC assays were conducted as reported previously 22. A total of 90 HCC tissues were obtained from the Affiliated Hospital of Youjiang Medical College for Nationalities. The clinicopathological features of HCC patients are shown in Supplementary Table S4. All samples were obtained with informed consent. The study was approved by the Ethics Committee of Renji Hospital, Shanghai Jiao Tong University School of Medicine (KY2023-084-B). Briefly, the sections were deparaffinized with xylene and rehydrated through graded alcohol solutions. Antigen retrieval was performed by heating the sections in sodium citrate buffer (pH 6.0) at just below boiling temperature for 20 minutes in a microwave oven. After being washed three times with PBS, endogenous peroxidase activity was blocked by incubating the sections with 3% hydrogen peroxide for 10 minutes. Sections were then incubated overnight at 4 °C with primary antibodies. Following PBS washes, sections were incubated with horseradish peroxidase (HRP)-conjugated secondary antibody at 37 °C for 30 minutes, followed by incubation with diaminobenzidine (DAB) solution. Nuclei were counterstained with Mayer's hematoxylin.

### Immunofluorescence analysis via confocal imaging

Briefly, cells were grown on Lab-Tek chamber slides (Nunc), fixed with 4% paraformaldehyde in PBS for 30 minutes, and permeabilized with 0.1% Triton X-100 in PBS for 15 minutes. The slides were incubated with primary antibodies in blocking solution overnight at 4 °C in a humidified chamber. Subsequently, the slides were washed three times with PBS and incubated with Alexa Fluor 488-conjugated and Alexa Fluor 555-conjugated secondary antibodies and 4′, 6-diamidino-2-phenylindole (DAPI) in blocking solution for 30 minutes at 37 °C in a humidified chamber. Images were obtained using a Leica TCS SP8 confocal microscope (Leica, Microsystems). Information of the antibodies is listed in Supplementary Table S3.

### Mitochondrial DNA Quantification

The mitochondrial DNA (mtDNA) quantification assay was carried out as described 23. Briefly, genomic DNA was extracted from HCC cells using TIANamp Genomic DNA Kit (TIANGEN, China). Relative mtDNA copy number was measured by a quantitative real-time PCR using SYBR^®^ *Premix Ex Taq* (Takara, Dalian, China). The list of primers is provided in Supplementary Table S3.

### Oxygen consumption rate (OCR) determination

OCR was measured with a Seahorse XFe 96 Extracellular Flux Analyzer according to the manufacturer's instructions 24. Briefly, cells were seeded in an XFe 96 plate with complete DMEM medium for 24 hours. Before 1 hour detection, the medium was changed to Seahorse XF Base Medium. OCR was assessed by sequentially adding the following compounds: oligomycin (1.5 μmol/L), FCCP (1 μmol/L), and rotenone and antimycin A (each 0.5 μmol/L). The results were normalized to the number of cells in each plate determined at the time of measurement.

### Detection of ROS

Intracellular ROS levels were measured by staining the cells with the cell-permeable 2',7'-dichlorodihydrofluorescein diacetate (DCFH-DA). Cells were washed with PBS and incubated with 10 μmol/L DCFH-DA dissolved in DMEM without serum for 20 minutes. Following incubation, cells were washed with DMEM without serum. The fluorescence signal indicating ROS levels was measured by flow cytometry.

For the analysis of mitochondrial production of ROS, cells were stained with 2.5 μM MitoSOX Red (Thermo Fisher). Briefly, cells were resuspended in 100 μL of pre-warmed HBSS containing Ca^2+^ and Mg^2+^ and placed in a 96-well plate. Then, 100 μL of Mitosox red (20 μM) diluted in prewarmed HBSS was added to each well. Cells were incubated 20 minutes at 37 °C. After incubation, cells were washed twice with FACS buffer and then resuspended in 100 μL of FACS buffer before analysis by flow cytometry.

### Coimmunoprecipitation (Co-IP) assay

The cells were harvested in RIPA (Upstate, Biotechnology) lysis buffer containing protease inhibitors for 40 min on ice and centrifuged at 12,000 × g for 10 min. Protein A/G agarose beads were incubated with anti-SNX17 or anti-STAT3 antibody or negative control IgG overnight on an orbital shaker at 4 °C. The immune complex was precipitated with protein-A/G agarose, washed five times and analyzed by western blotting.

### Statistical analysis

All the data are presented as the means ± standard deviations (SDs). Unpaired *t* test was used to compare the means between two groups, and one-way ANOVA was used to compare the means among multiple groups. The Kaplan‒Meier method was used to plot survival curves, which were compared by the log-rank test.* p* < 0.05 was considered to indicate statistical significance.

## Results

### Elevated SNX17 expression predicts poor prognosis in HCC

**Figure 1:**
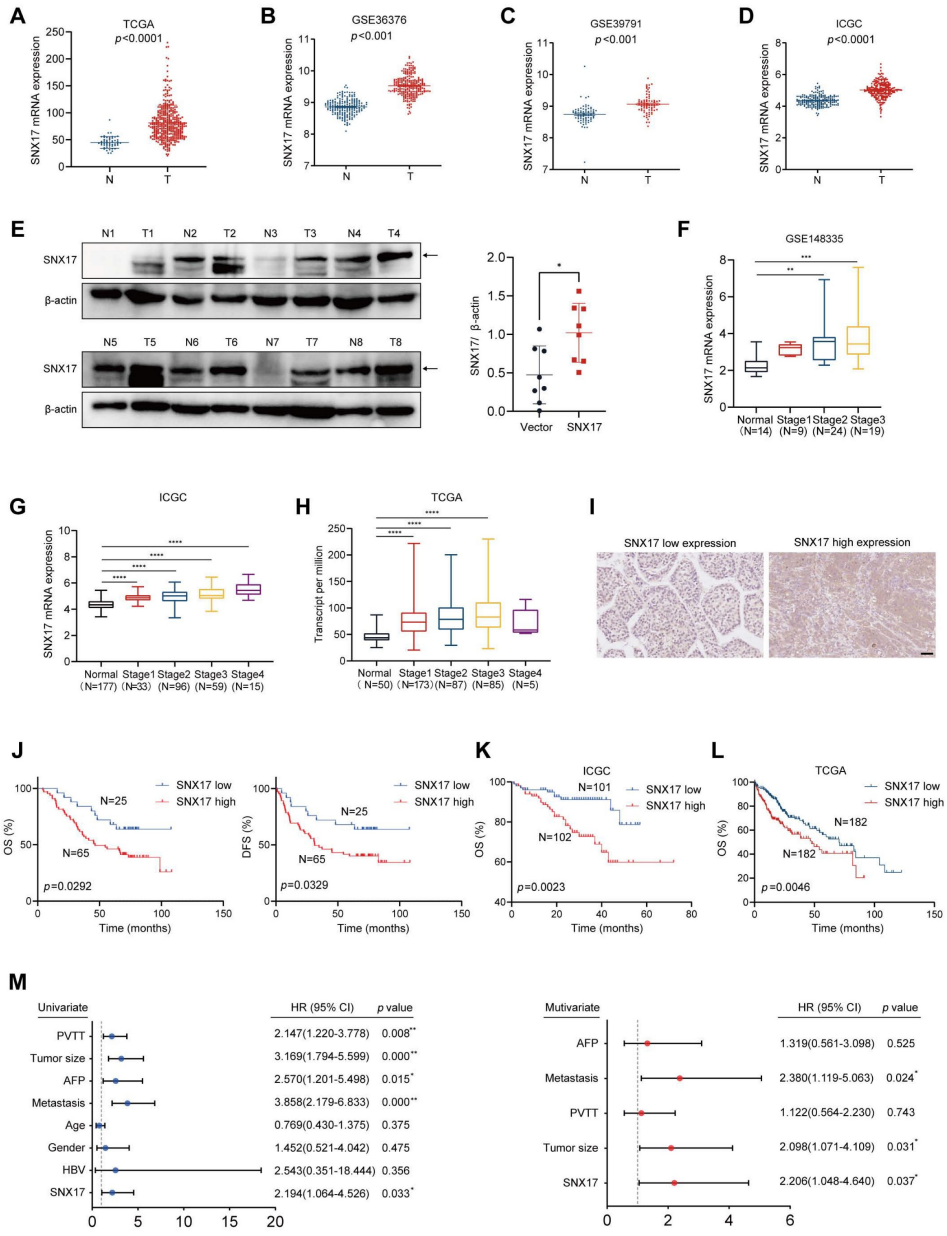
**Overexpression of SNX17 is associated with a poor prognosis in HCC patients.** (A-D) The expression of SNX17 in HCC tissues was compared with that in the corresponding noncancerous liver tissues in the TCGA datasets (A) (n = 50), the GSE36376 (B), GSE39791 (C) and the ICGC-LIRI-JP dataset (D). (E) The expression of SNX17 was detected by Western blotting in HCC tissues and adjacent non-tumor tissues. The arrow indicates the correct size of the band. (F-H) The expression of SNX17 in HCC tissues of different grades was analyzed in the GSE148335 (F), ICGC-LIRI-JP cohorts (G) and TCGA (H). (I) Immunohistochemical analysis of SNX17 expression in HCC samples. Representative images of samples with high and low SNX17 expression. (J) Overall and disease-free survival analysis of 90 HCC patients stratified by the SNX17 expression level. (K-L) Overall survival analysis of HCC patients in the ICGC-LIRI-JP cohort (K) and TCGA cohort (L) stratified by the SNX17 expression. (M) Univariate and multivariate Cox proportional hazards analyses were conducted to evaluate the HR of SNX17 in terms of the overall survival of patients with HCC. *p < 0.05; **p < 0.01.

We first analyzed the expression of SNX17 using The Cancer Genome Atlas (TCGA), Gene Expression Omnibus (GEO) and JP Project from the International Cancer Genome Consortium (ICGC-LIRI-JP) data. We found that the expression level of SNX17 in HCC tissues was higher than that in noncancerous tissues (Figure 1A-1D). In addition, expression level of SNX17 was upregulated in a variety of human tumors (Supplementary Figure S1A). SNX17 protein levels were upregulated in HCC tissues compared with noncancerous tissues according to the Western blotting (Figure 1E). Subsequent investigation revealed that SNX17 was upregulated in patients with nodal metastasis, as well as in patients with a high pathological stage using GEO, ICGC-LIRI-JP and TCGA data (Figure 1F-1H, Supplementary Figure S2).

We next investigated the relationship between SNX17 expression and prognosis in HCC patients. We first assessed the expression of SNX17 in HCC patients using IHC. According to the IHC results, the patients were divided into two groups based on their SNX17 expression levels (Figure 1I). We found that SNX17 expression was positively associated with gender and recurrence of HCC (Supplementary Table S5). However, no significant correlations were observed between SNX17 expression and other clinicopathological factors including age, tumor size, TNM grade, cirrhosis status, serum alpha-fetoprotein (AFP) level, vessel carcinoma embolus (VCE), HBV positivity status, portal vein tumor thrombus (PVTT) and vessel carcinoma embolus (VCE), capsule status (Supplementary Table S5). Kaplan-Meier survival analysis revealed that high SNX17 expression was significantly associated with shorter overall survival (OS) and disease-free survival (DFS) compared to low SNX17 expression (Figure 1J).

To further validate these findings, we analyzed data from TCGA and the ICGC, which confirmed the prognostic significance of SNX17 expression in HCC (Figure 1K-1L). Moreover, univariate and multivariate Cox proportional hazard analyses indicated that high SNX17 expression was an independent predictor of worse survival outcomes in HCC patients compared to those with low SNX17 expression (Figure 1M). In addition, high expression of SNX17 was linked to shorter OS times in patients with various types of tumors according to TCGA data (Supplementary Figure S1B). Taken together, these findings indicate that high expression of SNX17 is associated with a poor prognosis in HCC patients. SNX17 might play an important role in promoting the malignant progression of HCC.

### SNX17 aggravates HCC cell proliferation

**Figure 2:**
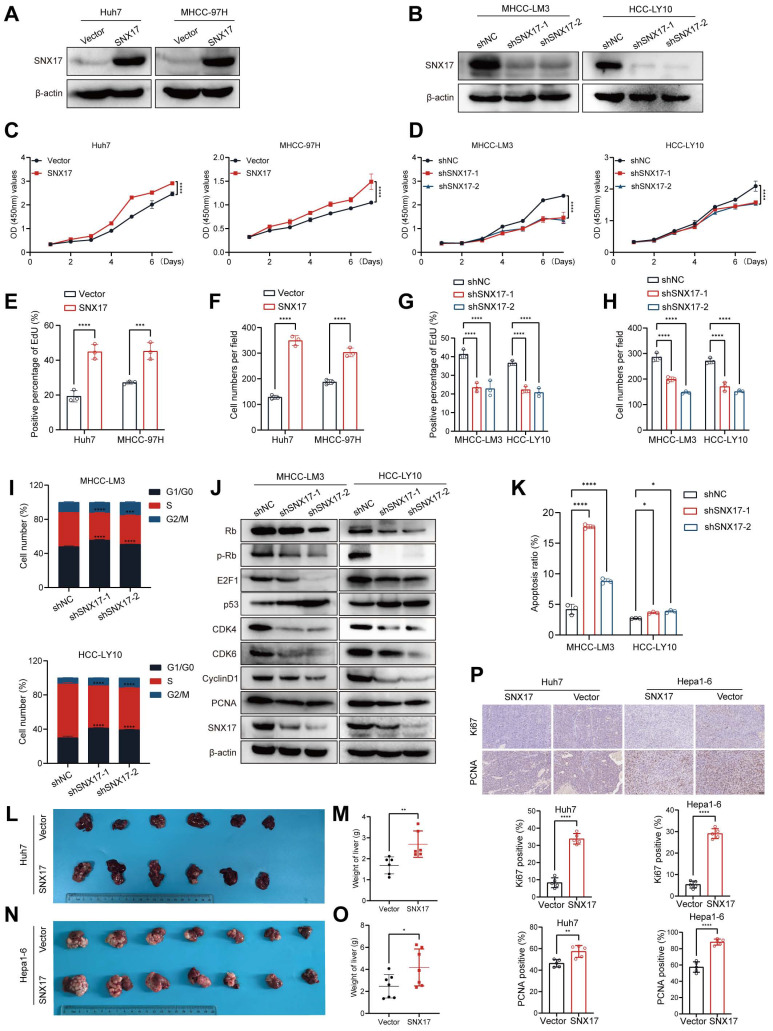
** SNX17 promotes HCC cell proliferation.** (A) SNX17 overexpression efficiency was validated using WB. (B) SNX17 shRNA knockdown efficiency was validated using WB. Analysis of the effect of SNX17 overexpression or knockdown on proliferation using CCK-8 assay (C, D) and EdU assay (E,G), and a colony formation assay (F,H). (I) The cell cycle distribution of cells was analyzed by flow cytometry. (J) The expression of cell cycle-related genes was detected by WB. (K) Apoptosis was analyzed by flow cytometry. (L-M) Liver tissues from animals bearing xenografts from Huh7 cells with stable SNX17 overexpression (L). The dot plots show the results of the quantitative analysis of liver weight (M). (N-O) Liver tissues from animals bearing isografts from Hepa1-6 cells with stable SNX17 overexpression (N). The dot plots show the results of the quantitative analysis of liver weight (O). (P) The expression of Ki67 and PCNA in xenograft tissues from Huh7 SNX17-overexpressing cells and isograft tissues from Hepa1-6 SNX17-overexpressing cells was evaluated by IHC. Bar = 50μm. *p < 0.05; **p < 0.01; ***p<0.001; ****p<0.0001.

To verify the function of SNX17 in HCC, we first examined the expression of SNX17 in HCC cell lines. The HCC-LY10, MHCC-LM3, Huh7 and MHCC-97H cells were selected for loss- or gain-of function studies due to their high or low endogenous SNX17 levels (Supplementary Figure S3A-S3B). We found that exogenous expression of SNX17 markedly promoted cell growth and colony formation ability. Conversely, SNX17 knockdown inhibited HCC cell proliferation and decreased the proportion of EdU-positive cells (Figure 2A-2H, Supplementary Figure S3C-S3E).

We also found that knockdown of SNX17 increased the proportion of cells entering the G1 phase and decreased the proportion of cells entering the S phase (Figure 2I, Supplementary Figure S4A). We next detected the expression of cell cycle regulators. The results showed that the levels of CDK4, CDK6, cyclin D1, Rb, p-Rb, E2F1 and PCNA were downregulated in SNX17-knockdown cells compared with control cells (Figure 2J). The expression of p53 was upregulated in SNX17-knockdown cells compared with control cells (Figure 2J). Furthermore, we found that knockdown of SNX17 induced apoptosis in HCC cells (Figure 2K, Supplementary Figure S4B). Therefore, these results suggest that SNX17 promotes the proliferation of HCC cells and inhibits their apoptosis. Therefore, these results suggest that SNX17 aggravates HCC cell proliferation.

To explore the effect of SNX17 on HCC tumorigenesis *in vivo*, we performed an orthotopic liver tumor model in nude mice. Compared with the control group, overexpression of SNX17 in Huh7 cells accelerated tumor growth (Figure 2L-2M). In addition, overexpression of SNX17 in Hepa1-6 mouse HCC syngeneic model promoted tumor growth (Figure 2N-2O, Supplementary Figure S5). Therefore, we speculate that SNX17 may affect the tumor microenvironment (TME). Macrophages are the most abundant innate immune cells in TME 25. M2 macrophages can secrete a variety of immunosuppressive factors, cytokines, and growth factors, which collectively contribute to an immunosuppressive tumor microenvironment. We next analyzed the relationship between SNX17 and M2 macrophages in HCC using TCGA data. We found that expression of SNX17 was associated with M2 macrophages infiltration. In addition, expression of SNX17 was associated with infiltration of myeloid-derived suppressor cells (MDSCs) and regulatory T cells (Tregs). Furthermore, there is a significant correlation between SNX17 and immunoregulator molecules (IL-10, TGF-β, CD163, PD-1 and PD-L1) (Supplementary Figure S6). Therefore, we speculate that SNX17 may influence the tumor suppressive microenvironment by regulating the infiltration of M2 macrophages, MDSCs, and Treg cells. Furthermore, the expression of Ki-67 and PCNA was significantly increased in HCC cells overexpressing SNX17 tumor tissues (Figure 2P). Taken together, these results provide strong evidence that SNX17 promotes the tumorigenesis of HCC.

### SNX17 promotes HCC cell migration, invasion and metastasis

**Figure 3:**
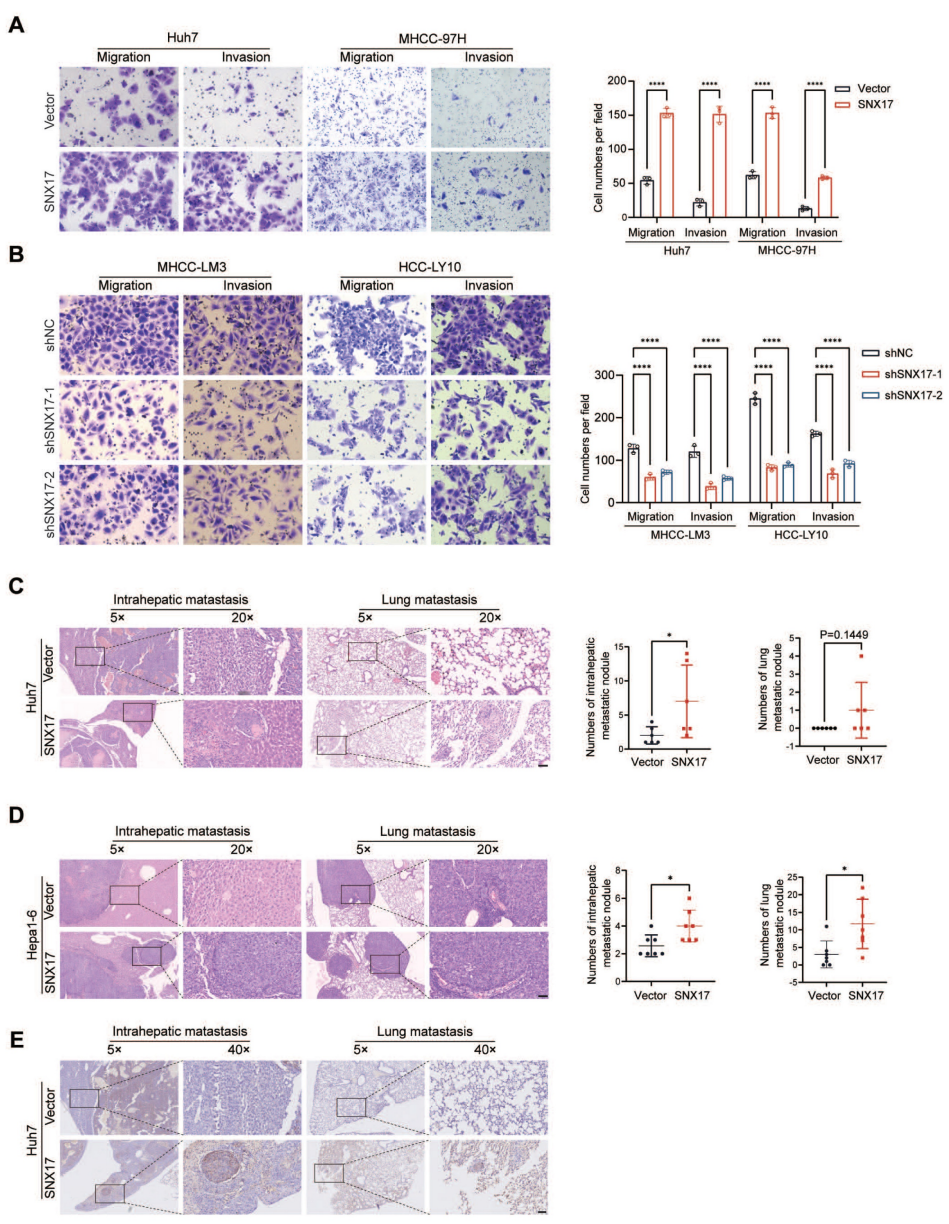
** SNX17 promotes HCC cell invasion and metastasis.** (A-B) The effects of SNX17 overexpression (A) and knockdown (B) on HCC cell migration and invasion were assessed by transwell assays. (C-D) Representative images of intrahepatic nodules and lung nodules formed by SNX17-overexpressing Huh7 (C) and Hepa1-6 (D) cells and control cells are shown. The numbers of intrahepatic metastatic nodules and lung metastatic nodules are shown in the right panel. Bar = 50 μm. (E) IHC analysis of metastasis using human-specific anti-mitochondria antibodies. *p < 0.05; ****p < 0.0001.

Since SNX17 was shown to be related to HCC metastasis (Supplementary Figure S2), we further examined the effect of SNX17 on the migration and invasion of HCC cells. We found that the overexpression of SNX17 promoted HCC cell migration and invasion, whereas silencing SNX17 mitigated cell migration and invasion (Figure 3A-3B).

To further clarify the role of SNX17 in HCC metastasis in vivo, SNX17-overexpressing Huh7 cells were orthotopically inoculated into the left hepatic lobe of mice via a microsyringe. Histological examination of lung and liver tissues indicated that the number of intrahepatic metastasis nodules was significantly higher in the SNX17 overexpression group compared with that in the control group (Figure 3C). However, the number of pulmonary metastatic nodules did not reach statistical significance. Subsequently, we performed orthotopic transplantation in C57BL/6 mice using Hepa1-6 cells overexpressing SNX17. The results revealed that overexpression of SNX17 significantly promoted the formation of intrahepatic metastasis nodules and pulmonary metastatic nodules (Figure 3D). Furthermore, metastasis was confirmed by anti-human mitochondria antibody staining, which is used to detect human cells in xenograft models (Figure 3E). Taken together, these findings indicated that SNX17 promoted HCC metastasis.

### SNX17 promotes oxidative phosphorylation and mitochondrial biogenesis in HCC cells

**Figure 4:**
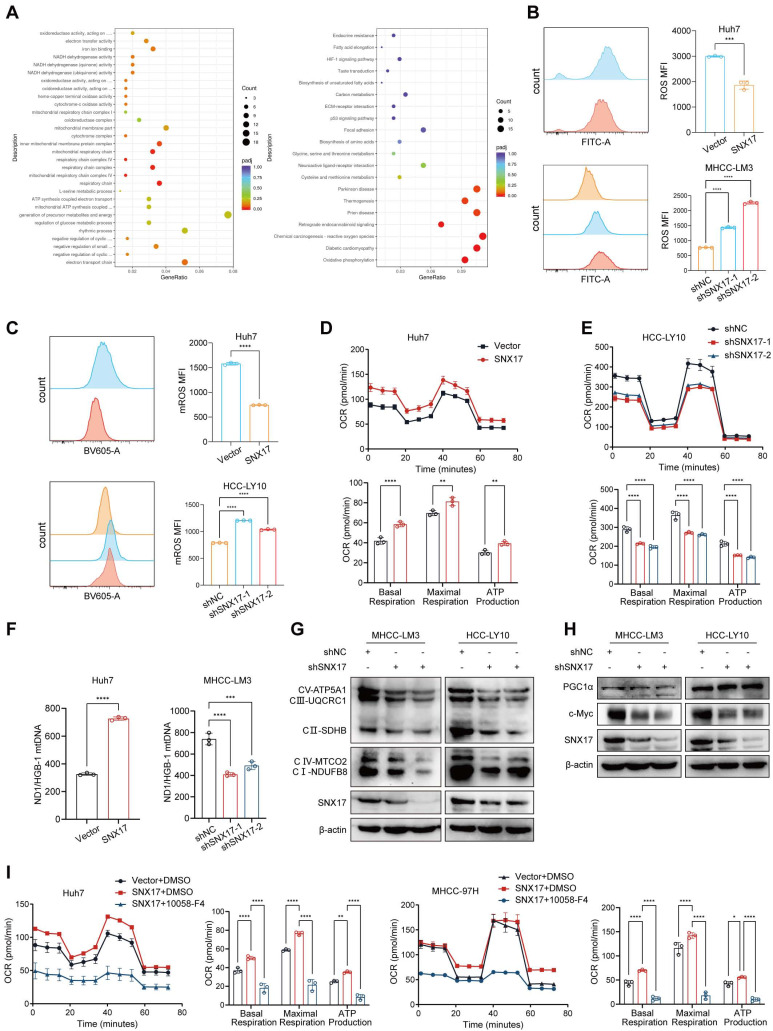
**SNX17 promotes mitochondrial biogenesis and oxidative phosphorylation through activation of c-Myc signaling**. (A) GO and KEGG pathway analysis based on SNX17 expression. (B) The level of ROS in SNX17 overexpression or knockdown cells was assessed by flow cytometry. (C) Mitochondrial ROS levels in cells stained with MitoSOX Red were evaluated by flow cytometry. (D-E) The OCR, basal mitochondrial respiration rate, maximal respiratory capacity, ATP production and spare respiratory capacity were calculated and statistically analyzed in SNX17 overexpressing HCC cells (D) and SNX17 knockdown HCC cells (E). (F) The content of mtDNA was measured by qPCR in SNX17 overexpression and knockdown HCC cells. (G) OXPHOS complexes were detected by WB in SNX17 knockdown HCC cells. (H) PGC-1α and c-Myc expression were examined by WB in SNX17 knockdown HCC cells. (I) The OCR, basal mitochondrial respiration rate, maximal respiratory capacity, ATP production and spare respiratory capacity were calculated and statistically analyzed in SNX17 overexpressing Huh7 and MHCC-97H cells treated with 10058-F4. *p < 0.05; **p < 0.01; ***p < 0.001; ****p < 0.0001.

To explore the mechanism of SNX17 in HCC, RNA sequencing was performed in SNX17-overexpressing cells. Many differentially expressed genes (DEGs) were identified in cells based on RNA-seq, and DEGs were further subjected to Gene Ontology (GO) and Kyoto Encyclopedia of Genes and Genomes (KEGG) enrichment analysis. The results revealed that SNX17 was involved in mitochondrial respiration and oxidative phosphorylation (Figure 4A). Furthermore, GSEA showed that SNX17 expression was positively correlated with genes related to "Mitochondrial gene expression" and "Oxidative phosphorylation" according to TCGA data (Supplementary Figure S7A). We examined the effects of SNX17 on intracellular ROS level in HCC cells. The results showed that overexpression of SNX17 decreased intracellular ROS level, while SNX17 knockdown markedly increased intracellular ROS level (Figure 4B). As mitochondria is a major source of ROS, we next examined ROS levels in mitochondria via MitoSox staining. The results showed that the mitochondrial superoxide anion level was significantly increased in SNX17-knockdown HCC cells, whereas mitochondria ROS was significantly decreased in SNX17 overexpression HCC cells (Figure 4C). We next determined the effects of SNX17 on mitochondrial metabolism by evaluating the oxygen consumption rate (OCR). The results showed that SNX17 overexpression significantly increased basal cellular respiration, intracellular ATP levels and maximal respiration, while SNX17 knockdown markedly suppressed the rate of oxygen consumption (Figure 4D-4E). Furthermore, the content of mitochondrial DNA (mtDNA) was markedly decreased or elevated when SNX17 was knocked down or overexpressed in HCC cells (Figure 4F). Moreover, overexpression of SNX17 leads to an increase in the expression of mitochondria-related genes (Supplementary Figure S7B). In addition, the expression of key mediators of OXPHOS, including NDUFB8, SDHB, UQCRC1, MTCO2 and ATP5A1 were markedly decreased or elevated in HCC cells when SNX17 was knockdown or overexpressed (Figure 4G, Supplementary Figure S8).

Given that PPARG, TFAM, PGC1α and c-Myc have been commonly recognized to play a central role in mitochondrial metabolism regulation 26-28. Consequently, we next investigated the effects of SNX17 on the expression of PPARG, TFAM, PGC1α and c-Myc. We found that SNX17 did not affect the expression of PPARG, TFAM and PGC1α (Supplementary Figure S9A). However, we found that overexpression of SNX17 increased c-Myc expression, while knockdown of SNX17 inhibited c-Myc expression (Figure 4H, Supplementary Figure S9B). To determine the cellular localization of c-Myc, we employed immunofluorescence staining. We found that overexpression of SNX17 increased the expression and nuclear localization of c-Myc in Huh7 cells (Supplementary Figure S10A). In addition, we found that overexpression of SNX17 increased the expression and nuclear localization of c-Myc in murine xenografts and allografts (Supplementary Figure S10B). Furthermore, GSEA analysis showed that the SNX17 expression was positively correlated with genes related to "Myc targets V1" and "Myc targets V2" using TCGA data (Supplementary Figure S7C).

Previous studies have shown that c-Myc directly regulates expression of genes involved in mitochondrial biogenesis and affects metabolic processes required for mitochondrial respiration 29. Therefore, we examined the effects of c-Myc on the content of mitochondrial DNA (mtDNA) and expression of key mediators of OXPHOS in HCC cells. We found that the content of mtDNA was markedly elevated when c-Myc was overexpressed in HCC cells. In addition, the expression of key mediators of OXPHOS, including NDUFB8, SDHB, UQCRC1, MTCO2 and ATP5A1 were markedly elevated in HCC cells when c-Myc was overexpressed (Supplementary Figure S11). Therefore, we consider that c-Myc induces mitochondrial OXPHOS through mitochondrial biogenesis.

To confirm the role of c-Myc in SNX17-mediated mitochondrial metabolism, c-Myc inhibitor 10058-F4 (Selleck, Houston, TX, USA) was used. The results showed that SNX17 overexpression-induced basal cellular respiration, intracellular ATP levels and maximal respiration were reversed by 10058-F4 (Figure 4I). Therefore, these results indicate that SNX17 promotes oxidative phosphorylation and mitochondrial biogenesis by regulating c-Myc in HCC cells.

### SNX17 interacts with STAT3 and increases phosphorylation of STAT3

**Figure 5:**
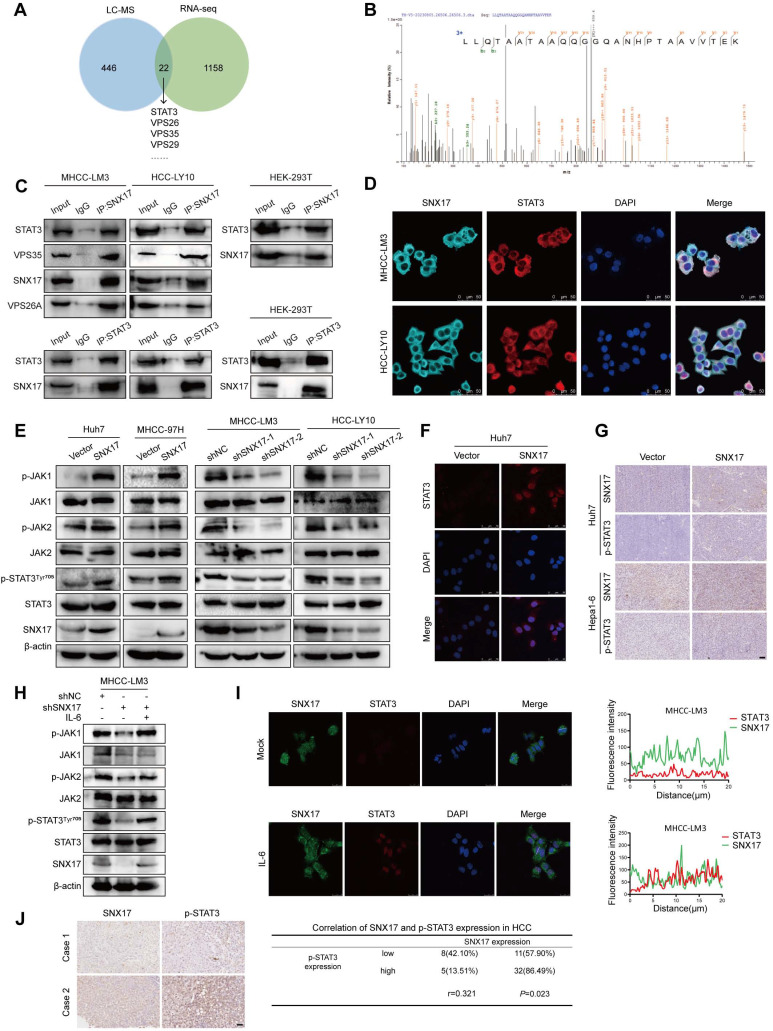
**SNX17-retromer interacts STAT3 and is associated with STAT3 activation.** (A) Venn diagram showing the overlap between SNX17-interacting proteins identified by LC-MS analysis and SNX17 RNA-seq datasets. (B) LC-MS spectrum of the peptide showing the sequence LLQTAATAAQQGGQANHPTAAVVTEK derived from STAT3. (C) SNX17 interacts with STAT3, VPS35, and VPS26 in HCC and 293T cells. (D) Colocalization of SNX17 and STAT3 in HCC cells. (E) The effects of SNX17 on the expression of p-STAT3 (Tyr705), p-Jak1, p-Jak2, STAT3, Jak1 and Jak2 in HCC cells. (F) STAT3 expression was evaluated by immunofluorescence staining. (G) The expression of SNX17 and p-STAT3 in xenograft tumor tissues from Huh7-SNX17 or isograft tumor tissues from Hepa1-6-SNX17 was detected by IHC. (H) The expression of p-STAT3, p-Jak1, p-Jak2, STAT3, Jak1 and Jak2 was examined by WB in SNX17 knockdown MHCC-LM3 cells treated with IL-6. (I) The expression of SNX17 and STAT3 was evaluated in MHCC-LM3 cells treated with IL-6 by immunofluorescence staining. (J) The correlation between SNX17 and p-STAT3 expression in HCC tissues was evaluated using IHC. Bar = 50 μm.

To explore the mechanism of SNX17 in HCC, we performed immunoprecipitation mass spectrometry (IP-MS) using an antibody against the V5 tag in total protein lysates from MHCC-97H cells overexpressing V5-SNX17. A total of 468 proteins were identified. Next, a total of 22 proteins were identified by integrating RNA-seq datasets and IP-MS datasets (Figure 5A). The retromer complex (VPS26, VPS35 and VPS29) was also found among 22 proteins demonstrating the reliability of IP-MS results. Furthermore, we found that overexpression of STAT3 increased c-Myc expression. Whereas, STAT3 knockdown inhibited c-Myc expression in MHCC-LM3 cells (Supplementary Figure S12D, S13). Our goal is focused on STAT3, a key transcription factor involved in various cellular processes including proliferation, apoptosis, angiogenesis, and immune regulation. IP assay results confirmed that SNX17 binds to STAT3 and retromer complex (VPS35 and VPS26A) (Figure 5B-5C). We identified STAT3 as a new interacting protein of SNX17 using Co-IP and immunofluorescence assay (Figure 5C-5D).

We next evaluated the effects of SNX17 on STAT3 phosphorylation. Our results showed that overexpression of SNX17 increased STAT3 phosphorylation, while knockdown of SNX17 inhibited STAT3 phosphorylation (Tyr705) (Figure 5E). Furthermore, SNX17 overexpression facilitated the nuclear entry of STAT3 in HCC cells (Figure 5F). The expression of p-STAT3 was increased in tumor tissues from HCC cells overexpressing SNX17 (Figure 5G). In addition, we found that Jak1/Jak2 was activated or inhibited in SNX17-overexpressing or SNX17-knockdown HCC cells (Figure 5E). Furthermore, we found IL-6 reversed the knockdown of SNX17-induced STAT3 suppression (Figure 5H). Interestingly, we also found that expression of SNX17 was increased in MHCC-LM3 and Huh7 cells stimulated with IL-6 (Supplementary Figure S12A-S12B). Furthermore, using immunofluorescence microscopy, we demonstrated that IL-6 treatment significantly promoted the colocalization of SNX17 and STAT3, indicating a potential increase in their interaction under these conditions (Figure 5I). Because the SNX17 promoter contains 2 putative STAT3-binding sites (JASPAR version 3.0), we speculated that STAT3 may directly bind to the SNX17 promoter (Supplementary Figure S12E-S12F). Furthermore, we found that overexpression of STAT3 increased SNX17 expression. Conversely, STAT3 knockdown inhibited SNX17 expression in MHCC-LM3 cells (Supplementary Figure S12C-S12D). We next assessed the relationship between SNX17 and STAT3 phosphorylation in human primary HCC tissues. Our results showed that there was a significant positive correlation between the expression of SNX17 and phosphorylation of STAT3 (Tyr705) in HCC tissues (Figure 5J). Furthermore, analysis of TCGA cohort showed that there was a significant positive correlation between the expression of STAT3 and SNX17 in HCC tissue (Supplementary Figure S14). Therefore, this may create a positive feedback loop between SNX17 and STAT3 which exacerbates malignant progression of HCC.

### SNX17-retromer complex acts as a platform for IL-6-induced STAT3 activation

**Figure 6:**
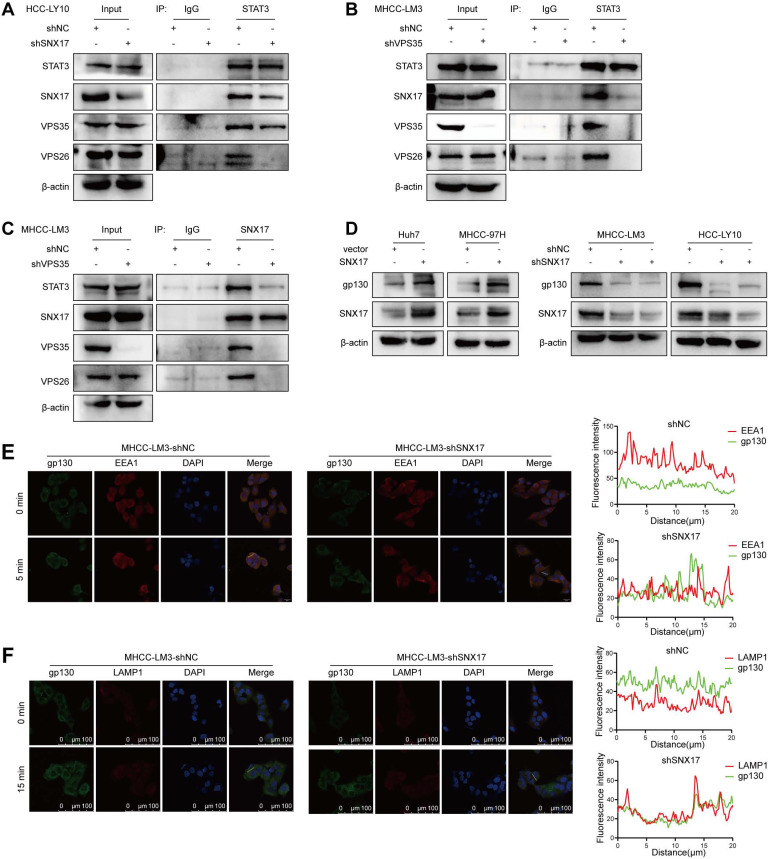
** SNX17 activates STAT3 in a retromer-dependent manner**. (A) Co-IP showed the effect of SNX17 knockdown on the interaction between SNX17 and STAT3. (B-C) Co-IP showed the effect of VPS35 knockdown on the interaction between SNX17 and STAT3. Immunocomplexes were analyzed with anti-STAT3 (B) or anti-SNX17 (C) antibodies. (D) The expression of gp130 was detected by Western blotting in SNX17-overexpressing and SNX17-knockdown HCC cells. (E-F) SNX17-knockdown MHCC-LM3 cells incubated with IL-6 for the indicated time periods were subjected to an immunofluorescence assay. Antibodies against gp130, EEA1 and LAMP1 were used.

The above results indicated that there was a direct interaction between SNX17 and STAT3. To further investigate this interaction, we performed knockdown experiments targeting SNX17. Our findings indicated that the knockdown of SNX17 significantly reduced the interaction between SNX17 and STAT3 (Figure 6A). VPS35 is a key component of the retromer complex. We then investigated whether VPS35 can play a vital role in SNX17-induced STAT3 activation. As shown in Figure 6B-6C, the binding capability between SNX17 and STAT3 was significantly decreased when VPS35 was knocked down in HCC cells. Binding of IL-6 to the IL-6R and the subsequent recruitment of gp130 leads to the activation of the JAK/STAT3 pathway 19. Previous studies showed that STAT3 activation depends on internalization of IL-6R and gp130 30. Therefore, we examined whether SNX17-mediated endocytosis was required for IL-6-induced STAT3 activation. We found that overexpression of SNX17 increased gp130 expression, while knockdown of SNX17 inhibited gp130 expression (Figure 6D). In addition, we found that SNX17 knockdown accelerated gp130 internalization. More gp130 predominantly colocalized with lysosomal degradation markers EEA1 and LAMP1 (Figure 6E-6F). Therefore, all results suggest that SNX17-retromer complex act as a platform for IL6-induced STAT3 activation in HCC cells.

### SNX17 promotes HCC cell proliferation, migration and invasion through the STAT3 pathway

**Figure 7:**
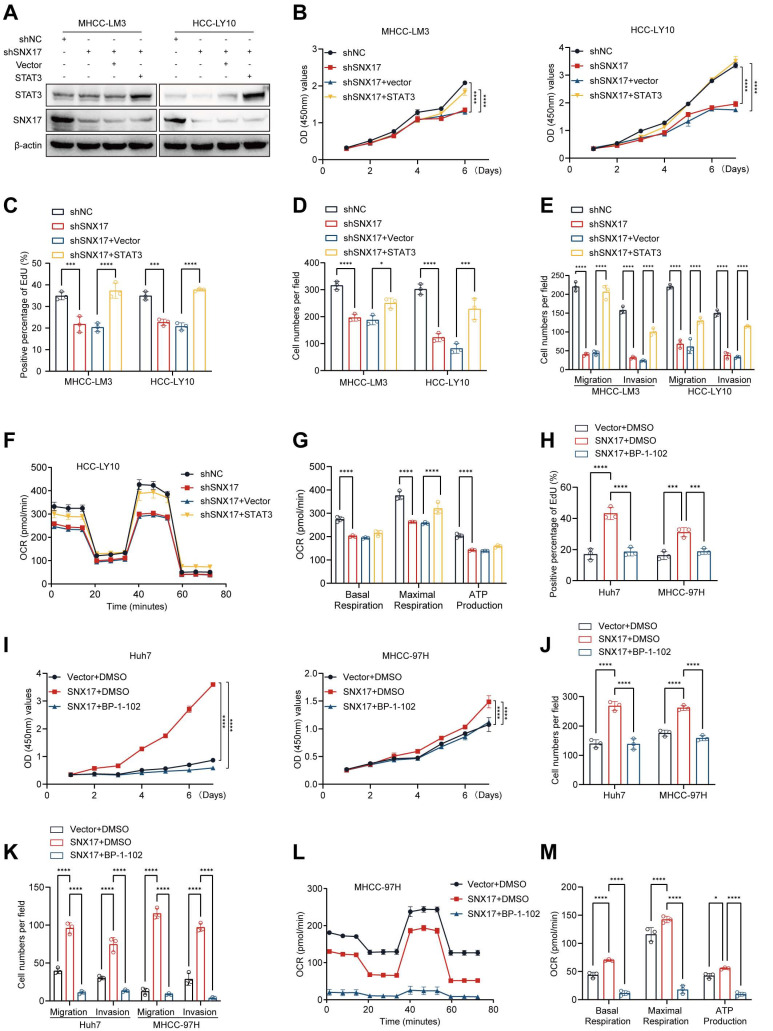
**SNX17 regulates HCC cell functions via STAT3 signaling**. (A) SNX17-knockdown HCC cells were transfected with STAT3, and the expression of SNX17 and STAT3 was detected by WB. (B-E) SNX17-knockdown HCC cells were transfected with STAT3 as indicated, and cell proliferation, migration, and invasion were evaluated by CCK-8 assay (B), EdU assay (C), colony formation (D), Transwell assays (E). (F-G) The OCR, basal mitochondrial respiration rate, maximal respiratory capacity, and ATP production were calculated and statistically analyzed in SNX17 knockdown HCC cells transfected with STAT3. (H-K) SNX17-overexpressing HCC cells were treated with BP-1-102 or DMSO as indicated, and cell proliferation, migration, and invasion were evaluated by EdU assay (H), CCK-8 assay (I), colony formation (J), and Transwell assays (K). (L-M) The OCR, basal mitochondrial respiration rate, maximal respiratory capacity, ATP production and spare respiratory capacity were calculated and statistically analyzed in SNX17 overexpressing HCC cells treated with BP-1-102. *p<0.05; ***p<0.001; ****p<0.0001.

To explore the role of STAT3 in SNX17-mediated HCC proliferation and invasion, SNX17-knockdown HCC cells were transfected with STAT3 plasmid. We found that SNX17 knockdown-induced suppression of cell proliferation, migration and invasion could be reversed by overexpressing STAT3 in HCC cells (Figure 7A-7E, Supplementary Figure S15A-S15C, Figure S15G). Furthermore, SNX17 knockdown-induced the inhibition of oxidative phosphorylation was reversed by overexpressing STAT3 in HCC cells (Figure 7F-7G). Conversely, SNX17 overexpression-induced promotion of cell proliferation, migration and invasion could be attenuated by STAT3 inhibitor BP-1-102 in HCC cells (Figure 7H-7K, Supplementary Figure S15D-S15F, Figure S15H). In addition, BP-1-102 significantly attenuated the promotive effect of SNX17 overexpression on the OCR, basal cellular respiration, intracellular ATP levels and maximal respiration in HCC cells (Figure 7L-7M). Therefore, these results suggested that SNX17 promotes cell proliferation and invasion through the STAT3 pathway in HCC cells.

## Discussion

Endocytosis regulates internalization, recycling and degradation of receptors which alter cancer cell invasion or metastasis 9. Multiple endocytic proteins are dysregulated in various cancers and play significant roles in tumor metastasis 8. SNXs associate with the retromer complex and mediate endosomal trafficking pathways 31. Among these, SNX17 has been implicated in the pathogenesis of several diseases, including cardiac diseases 16,32 and neurological disorders 17,33. Previous studies have shown that ITGB5 promotes SNX17-mediated endosomal recycling of transforming growth factor beta receptor type 2 (TGFBR2), which enhances gastric cancer metastasis 34. However, the role and underlying mechanisms of SNX17 in human cancer, including HCC, remain poorly understood, and the function of SNX17 has not been fully elucidated. In this study, we found that expression of SNX17 was upregulated and associated with a poor prognosis of HCC patients. SNX17 promotes HCC cell proliferation and migration. Given that SNXs can interact with the retromer complex to determine cargo selection, and considering that retromer dysfunction is linked to numerous diseases, including cancer, we further investigated the involvement of VPS35, a key component of the retromer complex. Previous reports indicate that VPS35 contributes to gastric cancer and HCC progression 35,36. SNX17 have been shown to be critical for recycling of numerous cell surface proteins 33,37. In this study, we found that knockdown of SNX17 impaired the recruitment of gp130, Jak1/2 and STAT3 to retromer. Furthermore, knockdown of VPS35 impaired the interaction between SNX17 and STAT3 in HCC cells. Previous study showed that cytoplasmic transport of STAT3 is an active process that requires receptor-mediated endocytosis 21. SNX17-retromer complex can act as a platform for IL6-induced STAT3 activation. Therefore, the SNX17-retromer complex may also provide a novel therapeutic target for HCC.

Persistent and aberrant activation of STAT3 is associated with inflammation and cancer development 19. Emerging evidence indicates that STAT3 acts as an oncogenic transcription factor, directly promoting the initiation and progression of HCC 38,39. Activated STAT3 can upregulate a variety of genes involved in cell growth including cyclins D1, Cdc25A, Cdc2, survivin and Mcl-1 40. Importantly, overexpression of STAT3 in tumor tissue has been found to be correlated with poor prognosis in HCC patients 41. In this study, we found that SNX17 promoted STAT3 activation and nuclear localization. The effects of SNX17 on promoting proliferation, migration and invasion was reversed by either STAT3 shRNA or a STAT3 inhibitor. Therefore, these data indicates that SNX17 promotes HCC progression through STAT3-mediated signaling. In addition, an interesting phenomenon was found. IL-6, a known activator of STAT3, induced SNX17 expression in HCC cells. Moreover, overexpression of STAT3 also increased SNX17 levels in HCC cells. Importantly, a significant positive correlation between SNX17 and STAT3 expression was observed in HCC tissues. Further analysis revealed a binding site for STAT3 within the SNX17 promoter region, suggesting direct transcriptional regulation of SNX17 by STAT3. Therefore, SNX17 and STAT3 can form a positive regulatory loop which promotes HCC malignant progression.

Macrophages are the most abundant innate immune cells in TME 25. Tumor-associated macrophages (TAMs), which are considered to be M2-like, exist in the TME and influence the metastasis of various cancers by interacting with cancer cells 42,43. M2 macrophages can secrete a variety of immunosuppressive factors, cytokines, and growth factors, which collectively contribute to an immunosuppressive tumor microenvironment**.** Immunoregulatory molecules, such as IL-10 and TGF-β, can induce M2 polarization 44. TAMs induce immunosuppression through expressing inhibitory immune checkpoints such as PD-1 and PD-L1, as well as by recruiting immunosuppressive cells such as Tregs 45. In this study, we found that overexpression of SNX17 significantly promoted the tumor growth in immunocompetent C57BL/6 mice. Furthermore, we found that expression of SNX17 was associated with M2 macrophages infiltration. In addition, expression of SNX17 was associated with infiltration of MDSCs and Tregs. In addition, there is a significant correlation between SNX17 and immunoregulator molecules (IL-10, TGF-β, CD163, PD-1 and PD-L1). Therefore, we speculate that SNX17 may influence the tumor suppressive microenvironment by regulating the infiltration of M2 macrophages, MDSCs, and Treg cells. The detailed mechanism requires further in-depth research to be fully elucidated.

**Figure 8:**
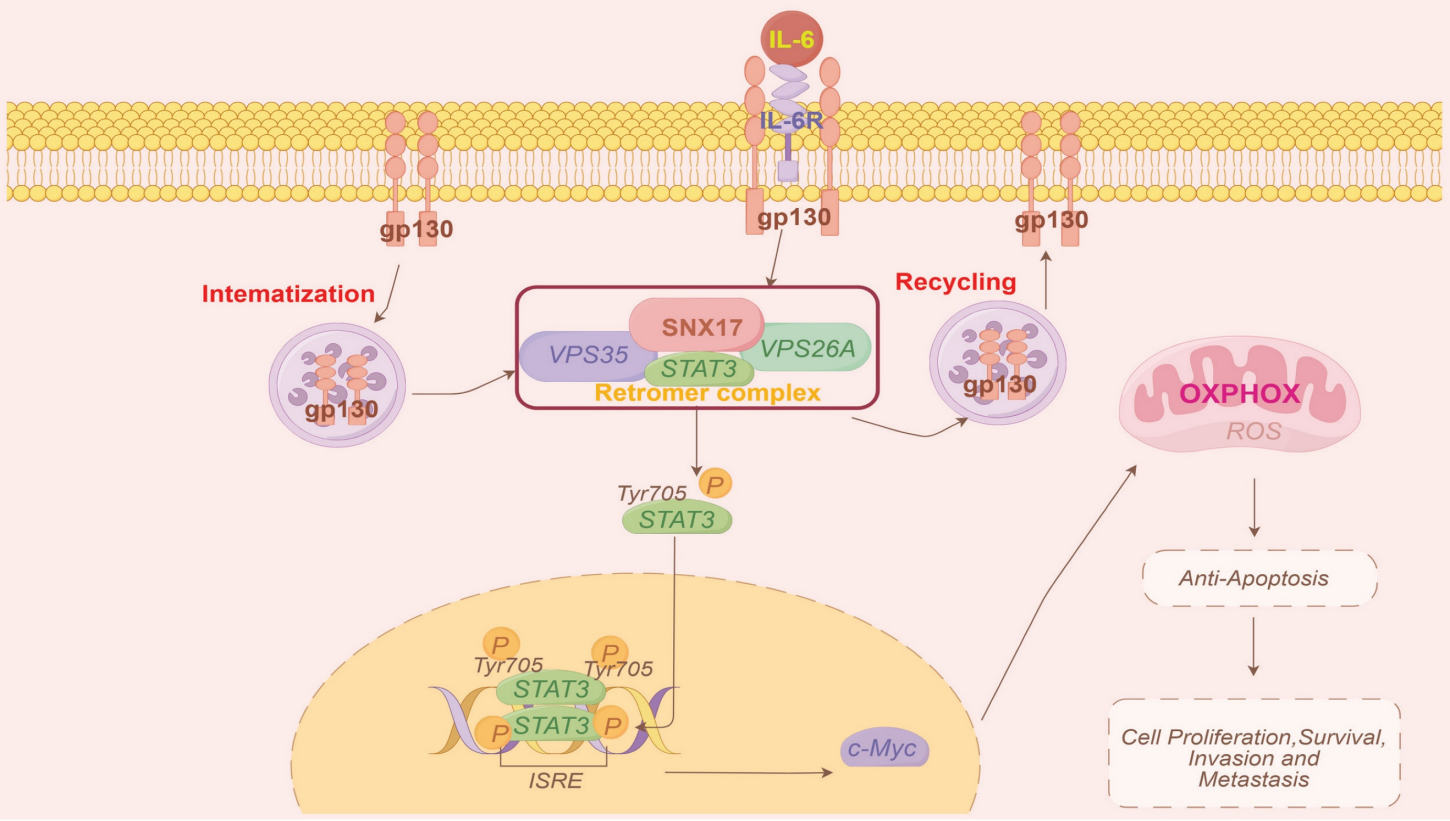
Model of the mechanisms of action of SNX17 in HCC. IL-6 induces SNX17 expression in HCC cells. The overexpression of SNX17 influences gp130 endosomal trafficking and increases STAT3 phosphorylation in a retromer-dependent manner, subsequently activates c-Myc and increases OXPHOS and promotes HCC progression.

In conclusion, our findings demonstrated that SNX17 upregulation was associated with a poor prognosis in HCC patients. SNX17 promoted HCC cell proliferation and metastasis via direct interaction with STAT3 in a retromer-dependent manner, subsequently increasing c-Myc expression and influencing OXPHOS and mitochondrial biogenesis (Figure 8). Our study elucidates the molecular mechanisms by which SNX17 contributes to HCC progression, highlighting its potential as both a prognostic marker and a therapeutic target.

## Supplementary Material

Supplementary figures and tables.

## Abbreviations

HCC: Hepatocellular carcinoma
TCGA: The Cancer Genome Atlas
GEO: Gene Expression Omnibus
ICGC-LIRI-JP: JP Project from International Cancer Genome Consortium
SNX17: Sorting nexin 17
STAT3: signal transducer and activator of transcription 3
PI: Propidium iodide
DMEM: Dulbecco's modified Eagle's medium
IHC: Immunohistochemistry
Co-IP: Coimmunoprecipitation
OCR: oxygen consumption rate
OXPHOS: oxidative phosphorylation
EEA1: Early endosome antigen 1
LAMP1: Lysosomal Associated Membrane Protein 1
PVTT: Portal vein tumor thrombus
VCE: Vessel carcinoma embolus
mtDNA: Mitochondrial DNA
GO: Gene Ontology
KEGG: Kyoto Encyclopedia of Genes and Genomes
ETC: electron transport chain
DEGs: differentially expressed genes
OS: overall survival
DFS: disease-free survival
IP-MS: immunoprecipitation mass spectrometry
