# Supplementary material for: SNX17 mediates STAT3 activation to promote hepatocellular carcinoma progression via a retromer dependent mechanism
Source: Int J Biol Sci. 2025 Mar 31;21(6):2762–79. doi: 10.7150/ijbs.110506 (PMC12035908; doi:10.7150/ijbs.110506)
Supplement: Supplementary file 1 — Supplementary figures and tables. [file ijbsv21p2762s1.pdf]

SNX17 mediates STAT3 activation to promote hepatocellular carcinoma progression via a retromer dependent mechanism

Yuqi Liu[1], Wei Tian[1], Chao Ge[1], Canxue Zhang[1], Zhihong Huang[1], Chi Zhang[1], Yue Yang[1], Hua Tian[1,2,3*]

[1] State Key Laboratory of Systems Medicine for Cancer, Shanghai Cancer Institute, Renji Hospital, Shanghai Jiao Tong University School of Medicine, Shanghai, China

[2] Department of Pathology, The Affiliated Hospital of Youjiang Medical University for Nationalities, Baise 533000, China

[3] The Key Laboratory of Molecular Pathology (Hepatobiliary Diseases) of Guangxi, Baise 533000, China

[*]**Correspondence：** *Hua Tian, Ph.D.,* State Key Laboratory of Systems Medicine for Cancer*, Shanghai Cancer Institute, Renji Hospital, Shanghai Jiao Tong University School of Medicine, 25/Ln 2200, Xietu Road, Shanghai 200032, China. Tel/Fax: +86-21-64432140，E-mail:* htian@shsci.org

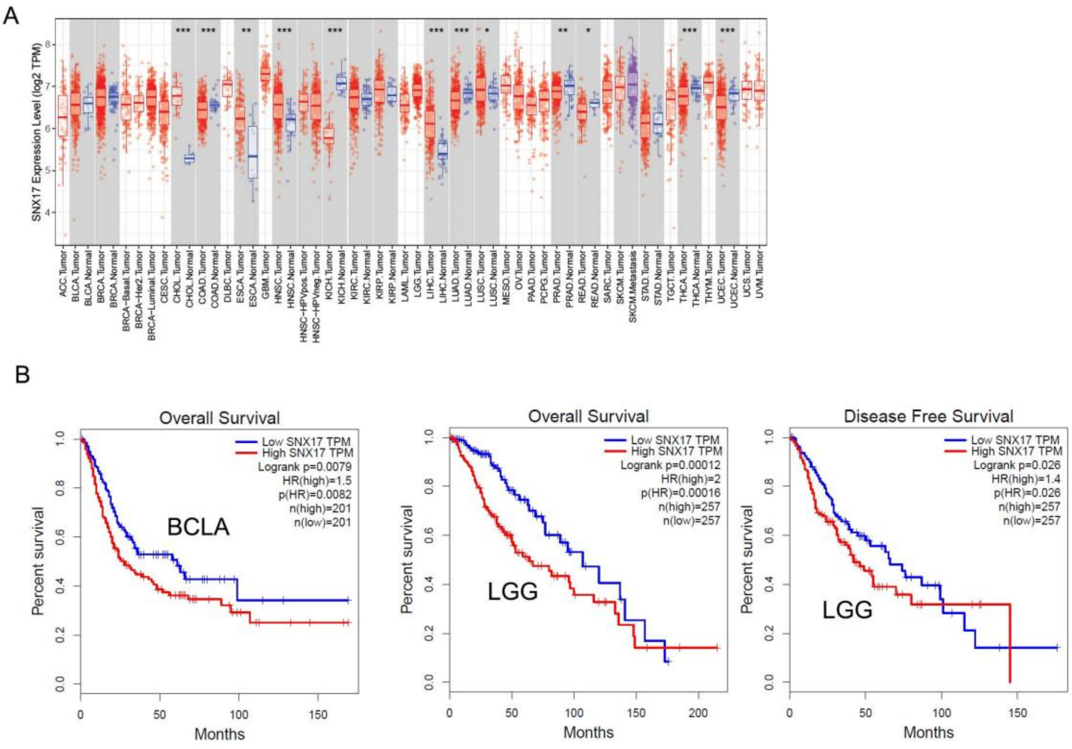

Supplementary Figure S1. (A) The expression of SNX17 in tumor tissues compared with paired corresponding noncancerous tissues was analyzed using data sets from TCGA. (B) Patients with high expression levels of SNX17 had shorter overall survival than patients with low expression levels, as determined using data sets from TCGA.

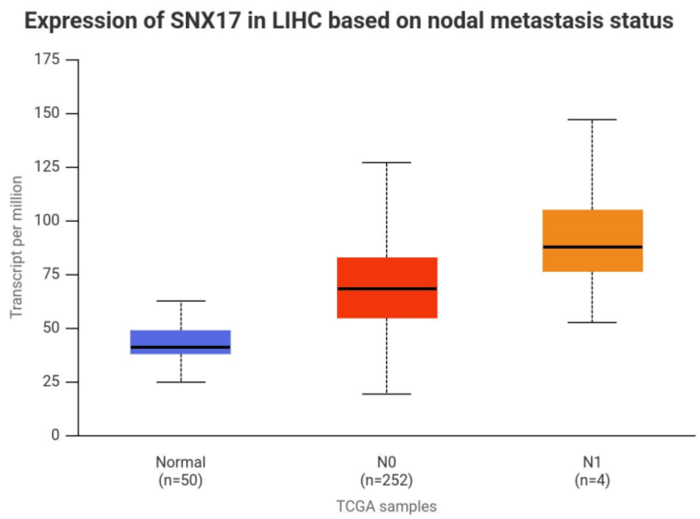

Supplementary Figure S2. SNX17 expression in noncancerous liver tissues, HCC tissues and metastatic cancer tissues was analyzed via TCGA data.

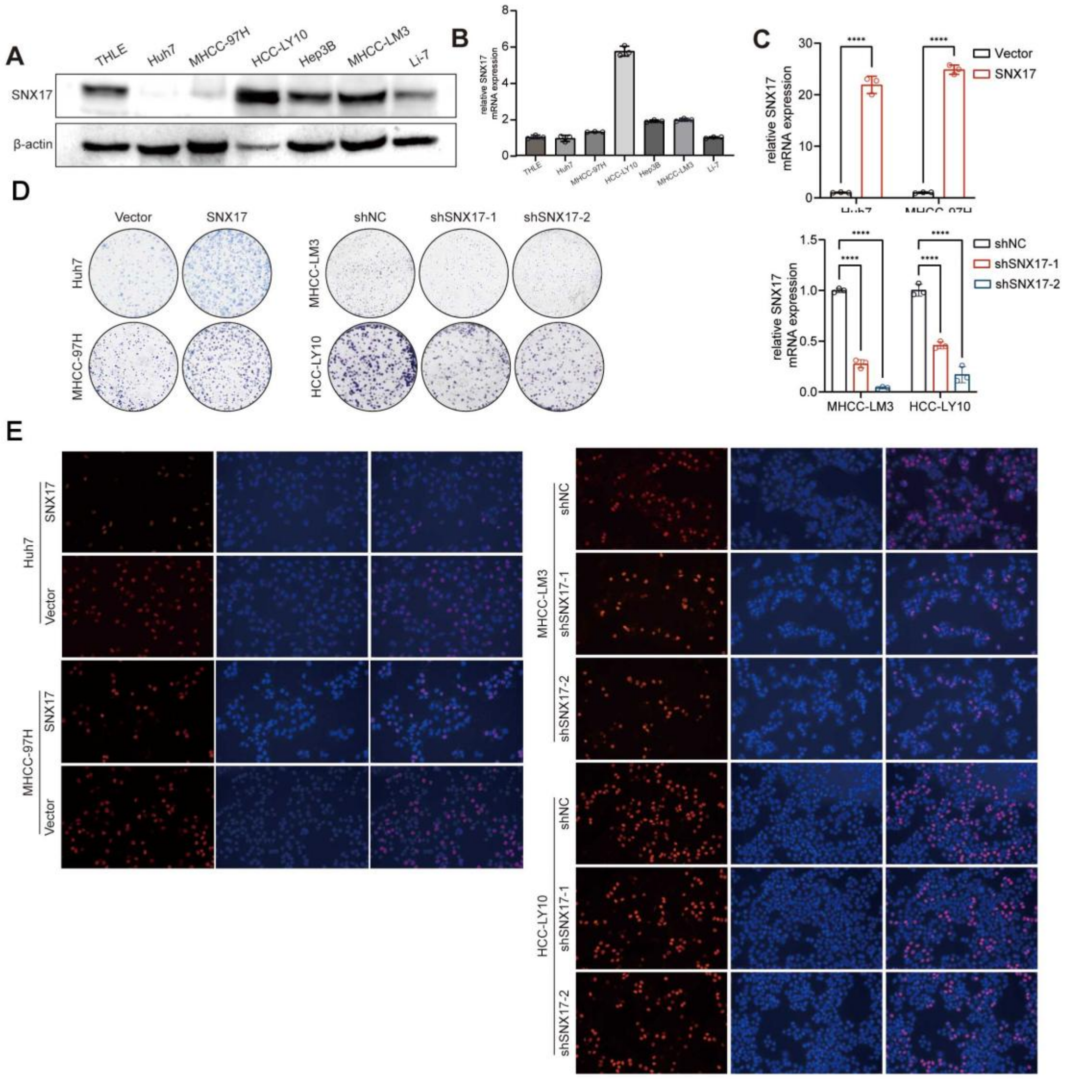

Supplementary Figure S3. (A-B) The expression of SNX17 was analyzed by Western blotting (A) and qRT-PCR (B) in HCC cell lines. (C) SNX17 overexpression and knockdown efficiency was validated using qRT-PCR. (D-E) The effect of SNX17 overexpression and knockdown on HCC cell proliferation was assessed by colony formation assays (D) and EdU assay (E).

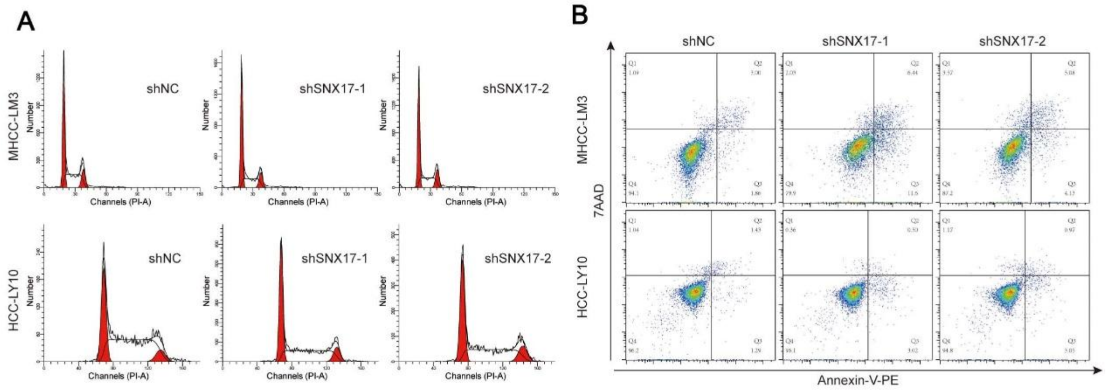

Supplementary Figure S4. (A) **T**he effect of SNX17 knockdown on HCC cell cycle was assessed by flow cytometry. (B) The effect of SNX17 knockdown on HCC cell apoptosis was assessed by flow cytometry.

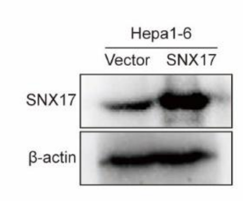

Supplementary Figure S5. SNX17 overexpression efficiency was validated using Western blotting in Hepa1-6 cells.

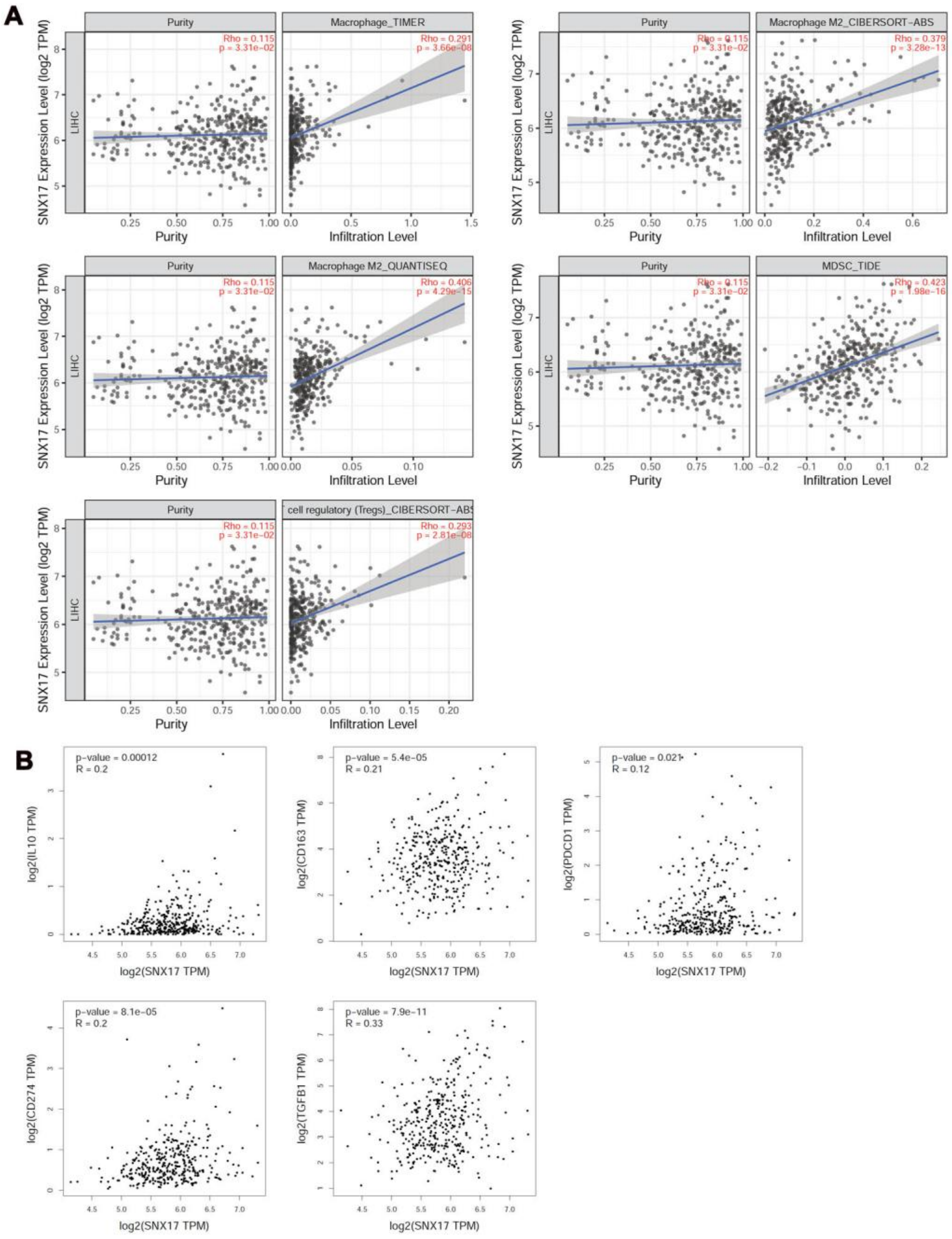

Supplementary Figure S6. SNX17 may affect the tumor microenvironment. (A) The relationship between SNX17 and immunosuppressive microenvironment in HCC was analyzed using TCGA data. (B) The relationship between SNX17 and immunoregulator molecules in HCC was analyzed using TCGA data.

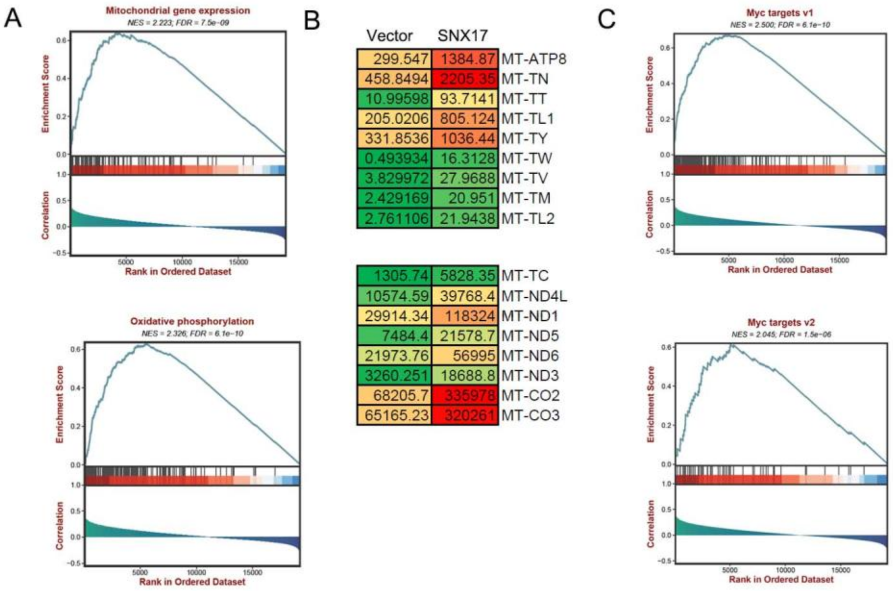

Supplementary Figure S7. (A) GSEA analysis showed that the SNX17 expression was positively correlated with genes related to “Mitochondrial gene expression” and “Oxidative phosphorylation”. (B) Heat map of differentially regulated genes in the mitochondrial gene set in vector and SNX17 overexpression HCC cells. (C) GSEA analysis showed that the SNX17 expression was positively correlated with genes related to “Myc target V1” and “Myc target V2”.

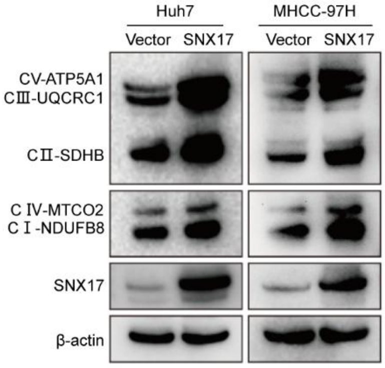

Supplementary Figure S8. OXPHOS complexes were detected by WB in SNX17 overexpression HCC cells.

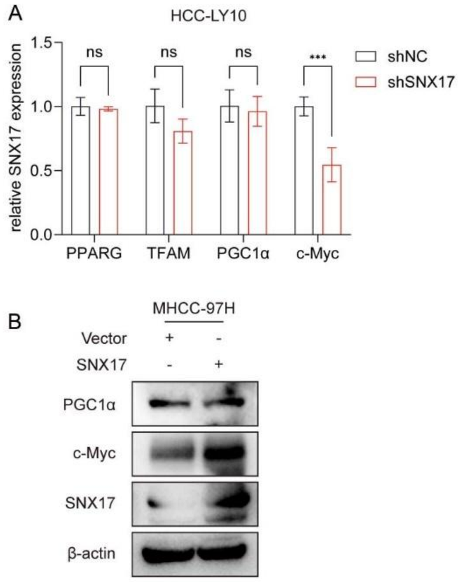

Supplementary Figure S9. (A) The expression of PPARG, TFAM, PGC-1α and c-Myc was assessed by qPCR in SNX17 knockdown HCC-LY10 cells. ns: not significant. ***P<0.001. (B) The expression of SNX17, PGC-1α and c-Myc was assessed by WB in SNX17 overexpression MHCC-97H cells.

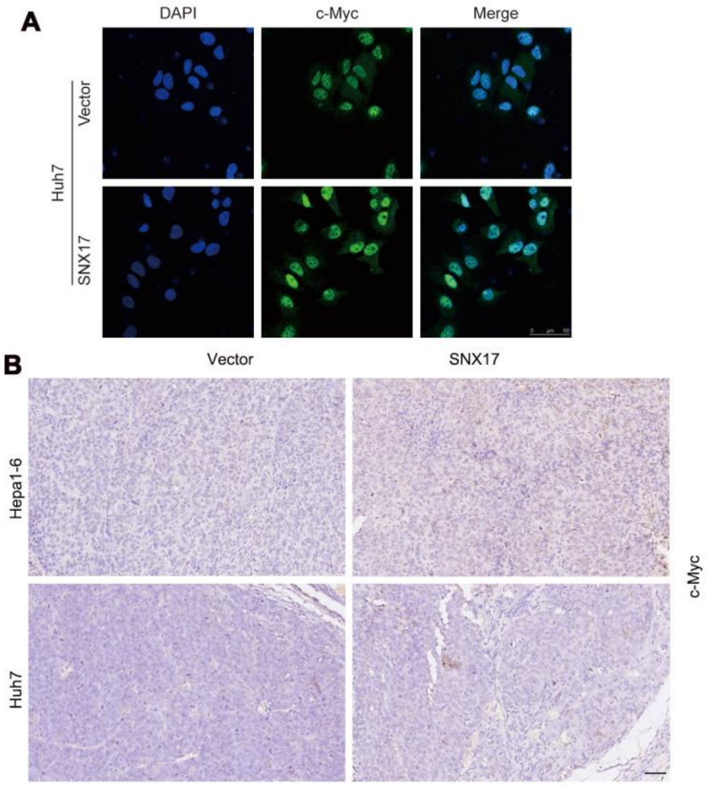

Supplementary Figure S10. SNX17 increased nuclear localization of c-myc in HCC cells. (A) The effects of SNX17 on the cellular localization of c-Myc in Huh7 cells. (B) The expression of c-Myc in xenograft tumor tissues from Huh7-SNX17 or isograft tumor tissues from Hepa1-6 SNX17 was detected by IHC. Bar=50 μM.

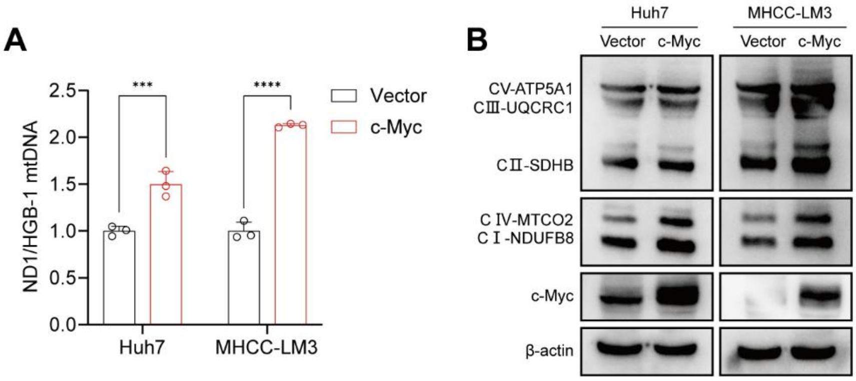

Supplementary Figure S11. The effects of c-Myc on the content of mtDNA and expression of OXPHOS complexes. (A) The content of mtDNA was measured by qPCR in c-Myc overexpression HCC cells. (B) OXPHOS complexes were detected by WB in c-Myc overexpression HCC cells. ***P<0.001; ****P<0.0001.

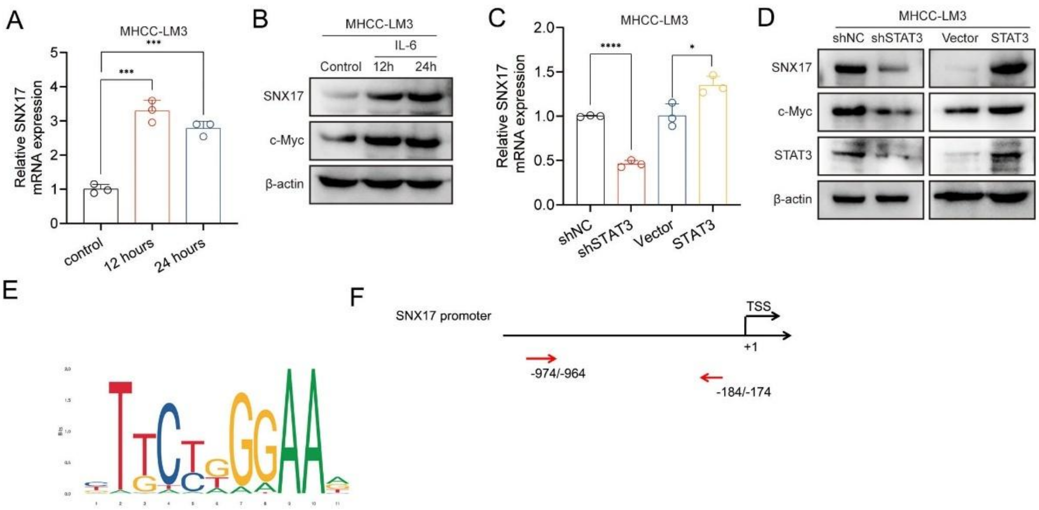

Supplementary Figure S12. (A) The expression of SNX17 was measured by qPCR in MHCC-LM3 cells treated with IL-6. (B) The expression of SNX17 and c-Myc was measured by WB in MHCC-LM3 cells treated with IL-6. (C) The expression of SNX17 was measured by qPCR in MHCC-LM3 transfected with STAT3 or shSTAT3. (D) The expression of SNX17 and c-Myc was measured by WB in MHCC-LM3 transfected with STAT3 or shSTAT3. (E) STAT3 binding motif. (F) Prediction of binding sites of STAT3 in the SNX17 promoter. *P<0.05; ***P<0.001; ****P<0.0001.

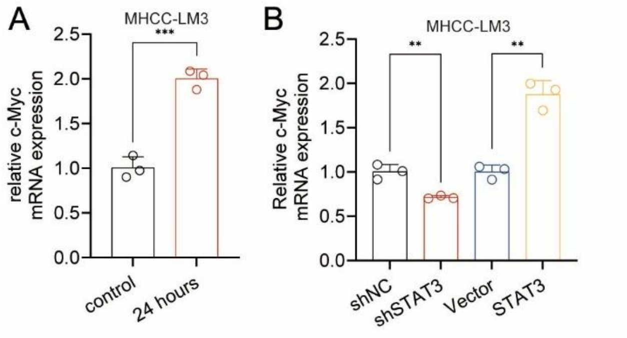

Supplementary Figure S13. (A) The expression of c-Myc was measured by qPCR in MHCC-LM3 cells treated with IL-6. (B) The expression of c-Myc was measured by qPCR in MHCC-LM3 cells transfected with STAT3 or shSTAT3. **P<0.01; ***P<0.001.

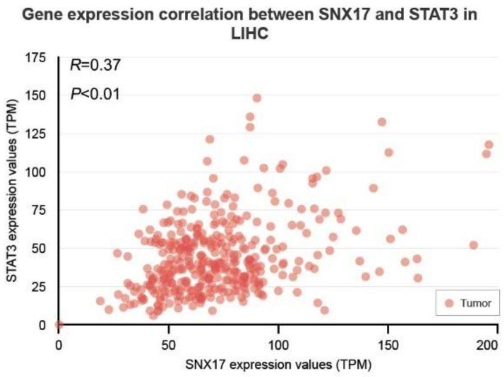

Supplementary Figure S14. The correlation between SNX17 and STAT3 using data sets from TCGA LIHC data.

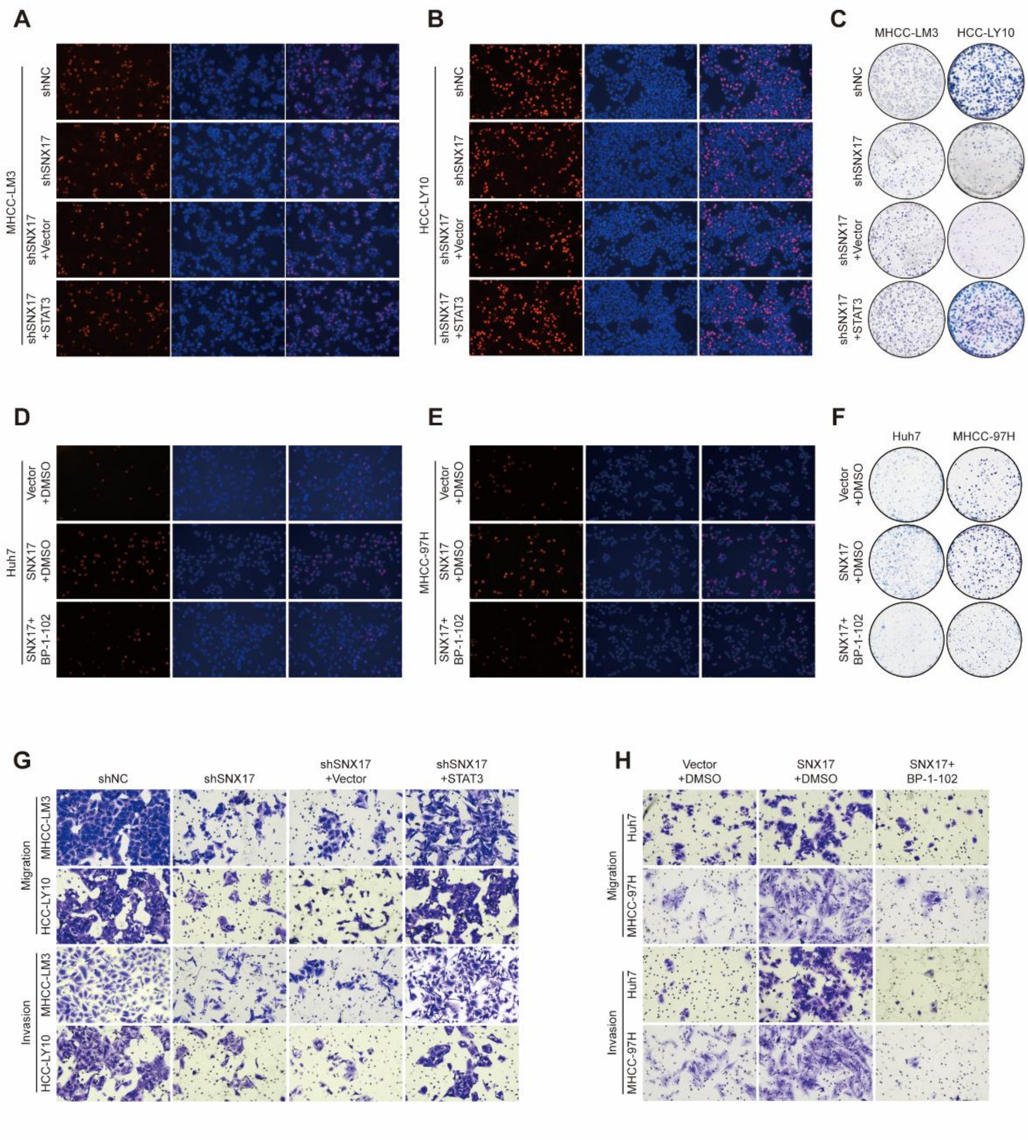

Supplementary Figure S15. SNX17 regulates HCC cell functions via the STAT3 pathway. (A-C) SNX17-knockdown HCC cells were transfected with STAT3 or vector. Cell proliferation was evaluated by EdU assay (A and B) and colony formation assays (C). Cell migration and invasion were evaluated by transwell assay (G). SNX17-overexpressing HCC cells were treated with BP-1-102 or DMSO as indicated. Cell proliferation was evaluated by EdU assay (D-E) and colony formation assays (F). Cell migration and invasion were evaluated by transwell assay (H).

Supplementary Table S1. The sequences of shRNA target

Identifier	Forward(5’-3’)
SNX17-1	GGCCTATAACATTCACGTGAAT
SNX17-2	GCAGCGAGACTTTCAACAGTTTC
VPS35	GGATATGGTACAACCCTTGT

Supplementary Table S2. The sequences of gene-specific primers used for qRT-PCR and vector constructs.

Gene name	Forward(5'-3')	Reverse (5'-3')
**Primers for qRT-PCR**		
SNX17	TCCTCTGTACCATTGCCCA	GCGCAGCATCTTCCTGATA
PGC-1α	CTCCCTGTGGATGAAGACGG	AGCTGAGTGTTGGCTGGTGC
ND1	CCCTAAAACCCGCCACATCT	GAGCGATGGTGAGAGCTAAGGT
HGB-1	GTGCACCTGACTCCTGAGGAGA	CCTTGATACCAACCTGCCCAG
PPARG	AGGATGCAAGGGTTTCTTCC	CTCTGGATTCAGCTGGTCGA
GAPDH	AGAAGGCTGGGGCTCATTTG	AGGGGCCATCCACAGTCTT C
TFAM	ATGGCGTTTCTCCGAAGC	TTTGCATCTGGGTTCTGAGC
**Primers for vector constructs**		
SNX17 (mouse)	CGACGCTCATGCACTTTTCCATTCCTGA	CGGAATTCTTACAGATCCTCATCTCCAA

Supplementary Table S3. Antibodies used in this study

Antibody	catalog	Dilution	Company
**For Western blotting**			
SNX17	10275-1-AP	1:500	Proteintech
CDK4	sc-56277	1:500	Santa Cruz
CDK6	sc-7961	1:500	Santa Cruz
Cyclin D1	sc-450	1:500	Santa Cruz
E2F1	sc-251	1:500	Santa Cruz
Rb	sc-102	1:500	Santa Cruz
p-Rb	CST-8516	1:500	CST
p-Jak2	cst-4406	1:500	CST
Jak2	cst-3230	1:500	CST
p-Jak1	cst-3331	1:500	CST
Jak1	CST-3344	1:500	CST
p-STAT3	CST-9145	1:500	CST
STAT3	10253-2-AP	1:500	Proteintech
P53	sc-126	1:500	Santa Cruz
PCNA	ab29	1:500	Abcam
gp130	67766-1-Ig	1:500	Proteintech
VPS26	12804-1-ap	1:500	Proteintech
VPS35	10236	1:500	Proteintech
PGC1α	sc-517380	1:500	Santa Cruz
c-Myc	10828-1	1:500	Proteintech
OXPHOS cocktail	PK30006	1:500	Proteintech
β-actin	A3854	1:20000	Sigma
Secondary antibody	HRP conjugated goat anti-rabbit IgG	1:4000	Proteintech
Secondary antibody	HRP conjugated goat anti-mouse IgG	1:4000	Proteintech
**For Immunohistochemistry**			
SNX17	PH4877	1:100	Abmart
Mitochondria	ab92824	1:1000	Abcam
Ki67	ab16667	1:500	Abcam
c-Myc	10828-1	1:100	Proteintech

PCNA	Ab29	1:2000	Abcam
Secondary antibody	Envision kit (HRP, rabbit/mouse, DAB+)	Ready-to-use	DAKO
**For Immunofluorescence staining**			
STAT3	10253-2-AP	1:50	Proteintech
gp130	a18036	1:50	Abclone
EEA1	66218-1	1:50	Proteintech
LAMP1	ab25630	1:50	Abcam
SNX17	10275-1-AP	1:50	Proteintech
c-Myc	10828-1	1:50	Proteintech
Secondary antibody	Alexa Fluor 555/488 anti-rabbit IgG	1:50	Invitrogen

Supplementary Table S4. Clinicopathological features of HCC patients (90 cases)

Clinicopathological features	Number
**Age**	
＜50	54
≥50	36
**Gender**	
Male	80
Female	10
**Tumor size**	
≤ 5 cm	62
>5 cm	28
**TNM grade**	
I- II	42
III- IV	48
**Cirrhosis**	
Negative	51
Positive	37
Missing	2
**AFP (ng/mL)**	
≤20	26
>20	63
Missing	1
**HBV**	
Negative	4
Positive	86
**Portal vein tumor thrombus (PVTT)**	
Negative	81
Positive	9
**Vessel carcinoma embolus (VCE)**	
Negative	59
Positive	31
**Capsule**	
Negative	31
Positive	59
**Tumor recurrence**	
Negative	49
Positive	41

Supplementary Table S5. Correlation between SNX17 levels in HCC patients and their clinicopathological characteristics

Clinicopathological features	Number	Low expression N (%)	High expression N (%)	*p* value
**Age**				
< 50	54	14(25.9)	40(74.1)	0.631
≥50	36	11(30.6)	25(69.4)	
**Gender**				
Male	80	19(23.8)	61 (76.2)	0.016*
Female	10	6(60)	6(40)	
**Tumor size**				
≤ 5 cm	62	17(27.4)	45(72.6)	0.910
>5 cm	28	8(28.6)	20(71.4)	
**TNM grade**				
I- II	42	13(31)	29(69)	0.529
III- IV	48	12(25)	36(75)	
**Cirrhosis**				
Negative	51	13(25.5)	38(74.5)	0.476
Positive	37	12(32.4)	25(67.6)	
**AFP (ng/mL)**				
≤20	26	9(34.6)	17(65.4)	0.379
>20	63	16(25.4)	47(74.6)	
**HBV**				
Negative	4	1(25)	3(75)	0.899
Positive	86	24(27.9)	62(72.1)	
**Portal vein tumor thrombus (PVTT)**				
Negative	81	24(29.6)	57(70.4)	0.239
Positive	9	1(11.1)	8(88.9)	
**Vessel carcinoma embolus (VCE)**				
Negative	59	18(30.5)	41(69.5)	0.425
Positive	31	7(22.6)	24(77.4)	
**Capsule**				
Negative	31	10(32.3)	21(67.7)	0.492
Positive	59	15(25.4)	44(74.6)	
**Tumor recurrence**				
Negative	49	18(36.7)	31(63.3)	0.038*
Positive	41	7(17.1)	34(82.9)	
